# Accelerated hit identification with target evaluation, deep learning and automated labs: prospective validation in IRAK1

**DOI:** 10.1186/s13321-024-00914-0

**Published:** 2024-11-14

**Authors:** Gintautas Kamuntavičius, Alvaro Prat, Tanya Paquet, Orestis Bastas, Hisham Abdel Aty, Qing Sun, Carsten B. Andersen, John Harman, Marc E. Siladi, Daniel R. Rines, Sarah J. L. Flatters, Roy Tal, Povilas Norvaišas

**Affiliations:** 1 AI Chemistry, Ro5, 2801 Gateway Drive, Irving, 75063 TX USA; 2 Strateos, 3565 Haven Ave Suite 3, Menlo Park, 94025 CA USA

**Keywords:** Computational chemistry, Artificial intelligence, Machine learning, Knowledge graph, Deep learning, Drug discovery, SBDD, MLSF, Docking, High-throughput screening, Hit identification, Virtual screening, Automated labs, IRAK1, Interleukin 1 receptor associated kinase

## Abstract

**Background:**

Target identification and hit identification can be transformed through the application of biomedical knowledge analysis, AI-driven virtual screening and robotic cloud lab systems. However there are few prospective studies that evaluate the efficacy of such integrated approaches.

**Results:**

We synergistically integrate our in-house-developed target evaluation (SpectraView) and deep-learning-driven virtual screening (HydraScreen) tools with an automated robotic cloud lab designed explicitly for ultra-high-throughput screening, enabling us to validate these platforms experimentally. By employing our target evaluation tool to select IRAK1 as the focal point of our investigation, we prospectively validate our structure-based deep learning model. We can identify 23.8% of all IRAK1 hits within the top 1% of ranked compounds. The model outperforms traditional virtual screening techniques and offers advanced features such as ligand pose confidence scoring. Simultaneously, we identify three potent (nanomolar) scaffolds from our compound library, 2 of which represent novel candidates for IRAK1 and hold promise for future development.

**Conclusion:**

This study provides compelling evidence for SpectraView and HydraScreen to provide a significant acceleration in the processes of target identification and hit discovery. By leveraging Ro5’s HydraScreen and Strateos’ automated labs in hit identification for IRAK1, we show how AI-driven virtual screening with HydraScreen could offer high hit discovery rates and reduce experimental costs.

**Scientific contribution:**

We present an innovative platform that leverages Knowledge graph-based biomedical data analytics and AI-driven virtual screening integrated with robotic cloud labs. Through an unbiased, prospective evaluation we show the reliability and robustness of HydraScreen in virtual and high-throughput screening for hit identification in IRAK1. Our platforms and innovative tools can expedite the early stages of drug discovery.

**Supplementary Information:**

The online version contains supplementary material available at 10.1186/s13321-024-00914-0.

## Introduction

Drug discovery is a notoriously lengthy, expensive and inefficient process [1]. Many of its major challenges and bottlenecks are now being tackled using modern data management [2], lab automation [3, 4] and machine learning (ML) [5–7] solutions that aim to transform the pharmaceutical industry’s legacy workflows [8]. Target identification and hit identification in the early stages of drug discovery are perfect examples of such transformations [8]. Traditionally, target identification has always been a largely manual process driven by experts with specialized domain-knowledge [9]. Recent advances in data management and analysis systems have enhanced researchers’ workflows, enabling seamless integration, summarization and retrieval of biomedical data to facilitate hypothesis generation. Examples of such systems include knowledge graphs [10] and platforms for target identification and evaluation [11]. Similarly, traditional high-throughput screening (HTS) methods for hit identification have been relying on slow and costly unguided experimentation platforms [12, 13]. In contrast, recently emerging automated robotic labs can now offer highly-reproducible data at greater throughput volume with better control of the experimental conditions [4, 14, 15].

Virtual screening for hit identification is one of the areas where ML, and in particular, deep learning (DL), techniques can now offer previously unattainable solutions and improved performance with respect to traditional alternatives [16]. Computational structure-based drug discovery (SBDD) techniques such as docking [17], and quantitative structure-activity relationship (QSAR) models [18], are now being augmented [19, 20] or complemented [21, 22] with these data-driven methods. A wide range of machine learning scoring functions (MLSF) are now available for application in virtual screening [16]. These methods are often extensively evaluated and compared using retrospective publicly available data [23]. However, their translation to practice is still limited with only a few prospective validation studies available [24], especially in comparison to the widely used computational chemistry techniques such as docking [13]. The impact of these methods in the real-world drug discovery programs will ultimately depend not only on their raw performance, as tested in benchmarking studies, but also on their ability to prioritize targets and compounds that could be brought to later stages of drug development.

In this study, we showcase an early-stage drug discovery workflow by integrating the Strateos robotic cloud labs for high-throughput screening with Ro5’s drug discovery suite, leveraging target evaluation (SpectraView)^1^ and DL-driven virtual screening (HydraScreen)^2^ [25] tools. We perform data-driven target evaluation and prospectively validate HydraScreen for virtual screening. Using the HTS results collected by the robotic cloud labs we also compare HydraScreen against traditional and machine learning, SBDD and QSAR techniques. Finally, we evaluate the identified hits in terms of their potential for further development.

## Methods

### Target evaluation using SpectraView

Target selection and evaluation was performed using SpectraView application. This tool allows data driven evaluation of prospective protein targets in drug discovery projects. The evaluation criteria encompass both scientific (e.g. biological, chemical) and commercial (e.g. novelty, competition) considerations, aligning with the typical questions posed by researchers in drug discovery campaigns. Results from these queries are presented as interactive plots that allow exploration of different criteria.

SpectraView draws all of its information for target evaluation from Ro5’s Knowledge Graph. The Knowledge Graph provides a comprehensive data resource consisting of four main components:

- Ontologies: databases containing entities with unique identifiers (e.g. Ensemble, HGNC, OpenTargets).
- Unstructured (textual) data: over 34 million PubMed abstracts and more than 90 million patents, from which we extract relevant entities and their relationships.
- Structured (database) data: 20 relational databases that provide contextual information for each entity type.
- Metadata and metrics: data origin metadata and custom metrics for data science analytics.

In total, the graph contains 12 entity types (Disease, Target, Mechanism, Compound, Species, Anatomical location, Cell line, Biomarker, Publication, Patent/Application, Author, Organization). Each entity is based on an ontology that provides unique identifiers for the associated concepts. For example, Disease and Target entities rely on the corresponding OpenTargets ontologies [26]. Entity-to-entity edges are extracted for all of these entity pairs. A Publication entity is introduced to preserve full contextual information when parsing text. As a result, conditional queries can be formulated for all combinations of extracted entities (e.g. Target—Diseases in the context of a Mechanism in a given Publication). Additionally, extensive metadata is extracted, including journal, author and organization affiliation. Corresponding entities (e.g. Author, Organization) are represented in the Knowledge Graph and are used in competitive landscape analyses. Finally, the Knowledge Graph is populated with metrics that allow quantitative analysis of the graph structure (e.g. network connectivity, point-wise mutual information, etc.) and entity relationship dynamics over time (e.g. edge emergence). Altogether, such detailed representation of entities and their relationships provide an in-depth and up-to-date data for drug discovery queries presented in SpectraView.

### Strateos cloud lab

All in vitro experiments were performed at the Strateos Cloud Lab in San Diego, CA. The Strateos Cloud Lab consists of a collection of online software applications that integrate Strateos’ automated chemistry and biology workstations, inventory management, data generation, and data management. All experiments are coded in autoprotocol (www.autoprotocol.org), an open-source standard developed by Strateos, which coordinates instrument actions in specific work cells based on scientific intent. This platform allows scientists to configure experiments and experimental parameters, remotely initiate and monitor automated experiments, oversee protocol management and inventory, generate data, and access real-time outputs of experimental data in a closed-loop fashion.

### 47k diversity library

A diverse library of 46,743 commercially available compounds was employed as the primary screening resource. This library was made from a broader pool of $\sim$500,000 compounds through cheminformatics evaluation. The chosen compounds were characterized by properties such as scaffold diversity and favorable physicochemical attributes. Compounds prone to interference were systematically removed, aligning with the exclusion of Pan Assay Interference Compounds (PAINS) from screening libraries. Compound stocks were stored at a concentration of 10 mM in dimethylsulfoxide (DMSO). For the screening process, 50 $\mu$L of each compound was dispensed into Echo-qualified 384-well polypropylene microplates. It is important to note that this 50 $\mu$L volume refers to the 10 mM stock compounds in the library plates, not the assay plates. These library plates were then used to create assay-ready plates, where 10 nL of each compound was transferred into screening plates using a Beckman Echo.

### 47k diversity library ligand preparation & stereoisomer treatment

The SMILES representations of the compounds in the 47k diversity set were processed by removing salts and converting them into a canonical form. Stereoisomers of the same compound were treated as different ligands in silico. For compounds with four or fewer undefined stereocenters, we generated and stored all possible stereoisomers, which amounted to a maximum of 16. For compounds with more than four stereocenters we randomly selected a subset of 16 stereoisomers to be used in virtual screening. Since empirical values collected from assays in vitro will correspond to racemic-averaged results, we compute a final per-compound score by averaging the scores across all stereoisomers in silico.

### HydraScreen

HydraScreen is a machine learning scoring function (MLSF) composed of a CNN-based (convolutional neural network) deep learning framework designed to predict protein-ligand affinity and pose confidence scores [25]. HydraScreen consists of an ensemble of models trained on more than 19K protein-ligand pairs and 290K docked conformations. It has been shown to outperform traditional SBDD and novel MLSFs solutions in both affinity and pose estimation tasks [25]. In this study, HydraScreen is employed to classify between strong and weak binders during virtual screening.

**Fig. 1:**
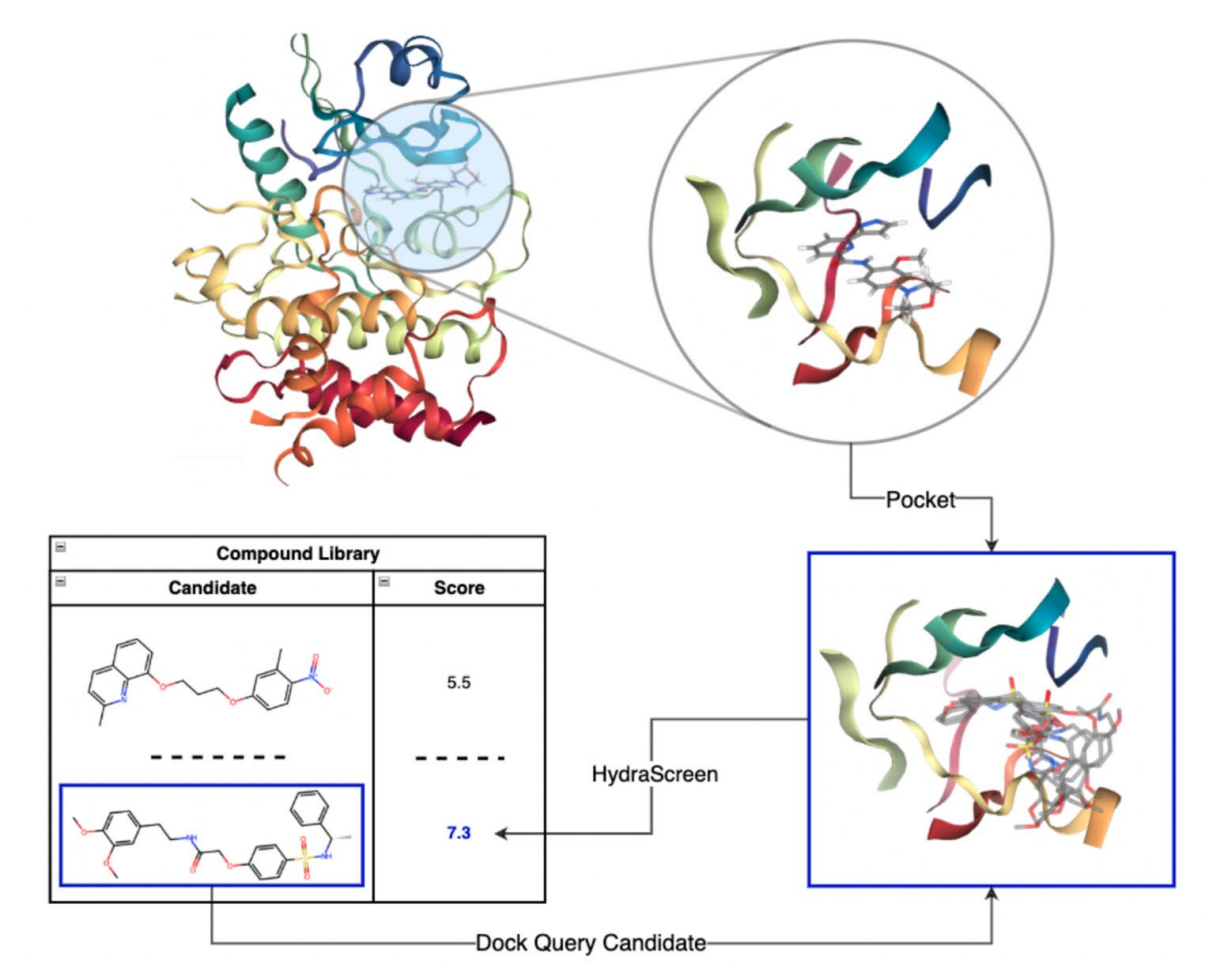
End-to-end structure-based scoring via HydraScreen. interleukin 1 receptor associated kinase 1 (IRAK1) crystal structure 6BFN and the associated ligand DL1 were used to define the pocket and relevant residues (top). For each compound in the library a pose ensemble was created via docking. The pose ensembles were then used as an input in HydraScreen to predict the compound affinity and pose confidence scores

HydraScreen estimates the affinity of a query ligand for a given target protein in a two-step process. First, it generates a set of conformations for protein-bound ligand, creating a docked pose ensemble. Second, it estimates the affinity and pose for each conformation and calculates a final aggregate affinity value using a Boltzmann-like average over the entire protein-ligand conformational space. A schematic of the described procedure is presented in Fig. 1.

Docked poses are generated in a similar fashion to that outlined in [25]. Briefly, we use the open-source Smina [17] software to generate poses of a query ligand in the binding pocket of our target protein. For each protein-ligand pair, the docking process involves: (1) preparation of the protein structure; (2) preparation of the ligand (candidate) structure; (3) docking with Smina. To prepare the protein for docking we perform a series of steps, including: (1) solvent and ion deletion, (2) repair of truncated side-chains using Dunbrack 2010 rotamer library [27], (3) adding hydrogens (histidines were treated like other standard residues), (4) adding charges. Additionally, non-standard residues were changed to the nearest standard residue. As an example, selenomethionine (MSE) is converted to methionine (MET).

Each ligand undergoes sanitization through RDKit (ver. 2021.09.03). Only 160 from the diversity set failed to sanitize and were thus excluded. An initial ligand conformer is generated with RDKit and undergoes protonation with the ADFR suite [28] at pH 7.4. For each compound, an initial conformer is then used to generate up to 20 docked poses via Smina, using the following input parameters: (num_modes = 20), (min_rmsd = 1Å). Furthermore, we define the binding pocket with the autobox option, passing in the reference crystal ligand pose (DL1) from 6BFN, and including all protein atoms within 4Å of any atom in the native ligand’s conformation. The ligand poses generated using this approach are available at https://ro5-public.s3.amazonaws.com/47k_poses.zip.

In this study, we primarily use HydraScreen to find potential hits amongst compounds in a screening library, therefore we rely on its ranking to identify compounds that successfully bind to the pocket above a given affinity threshold.

HydraScreen is available as an open-source Python package (https://pypi.org/project/hydrascreen/) free for non-commercial use and can be downloaded from PyPi package repository using *pip*.

### Benchmarks

We introduce a set of baselines consisting of structure-based and ligand-based methods to better understand the performance of HydraScreen with respect to traditional approaches.

***Smina***

Smina [17] exploits a traditional docking approach. Herein, protein-ligand binding affinity is scored according to the energy required to remove a ligand from the pocket (free energy). In order to score our compounds, we leverage the already generated poses and, for each docked ensemble, extract the largest free energy calculated by Smina amongst all the poses.

***DeCAF***

Density-Encoded Canonically Aligned Fingerprint (DeCAF) [29] is a ligand-based approach that measures the similarity between two compounds. DeCAF can be used to rank compounds by rewarding similarity between the query candidate and the reference molecule (DL1). DeCAF score is computed by: (i) finding the maximal common subgraph between the corresponding molecular graphs, represented as a coarse network of pharmacophore descriptors; (ii) computing the modular product of the two graphical models and extracting the similarity between the maximal clique identified. The score $\in [0,1]$ can then be used to rank compounds, where higher and lower scores correspond to a higher and lower structural pharmacophore match. In contrast to other shape-based methods like USRCAT [30], DeCAF does not require conformer generation.

***Random forest***

We trained a Random Forest (RF) classifier using publicly available IRAK1 data. The available pKi and pIC$_{50}$ values were converted from IRAK1 assays to boolean values based on whether they are above the $6.0 \ pIC_{50}$ threshold (sub-micromolar concentration). Out of 689 molecules available on PubChem, 142 were classified as active and 547 as inactive. The inactive class was further up-sampled by 5K using DeepCoy [31]. The compounds generated with DeepCoy were ensured to be structurally dissimilar to the actives while maintaining similar molecular weight as well as synthetic accessibility. By adding additional negative data, the models not only become harsher in inference by broadening the gap between active (1) and inactive (0) scores, but also become more robust to false positives. Since the ratio of active to inactive compounds in the training set is not representative of the typical ratio found in screening, we added additional data to reduce the model’s false positives. The classification model was trained using ECFP4 fingerprints [32] generated using RDKit.

***Pharmit***

Pharmit [33] provides an online, interactive environment for the virtual screening of large compound databases using pharmacophores, molecular shape and energy minimization. We used the co-crystallized structure 6BFN to extract a 6-point pharmacophore hypothesis, later used in scoring the 47k diversity set compounds. In order to create a continuous score that can be used to rank the compounds rather than a boolean match, we extended Pharmit’s compound and hypothesis matching functionality. The continuous score was computed by evaluating subsets of the original pharmacophore hypothesis, performing conformer matching on them and then combining results from the subset matches to get the final score. Such a hypothesis-subset screening was made possible by the high efficiency of the Pharmit algorithm.

### IRAK1 assay

The experimental method of LanthaScreen^TM^ Eu Kinase Binding Assay for IRAK1 was developed based on the Invitrogen^TM^ IRAK1-GST LanthaScreen^TM^ binding assay. Purified recombinant IRAK1-His (cat. # 40202) was purchased from BPS Bioscience Inc. (San Diego, CA, USA). Kinase tracer 236 (cat. # PR9078A) was purchased from Thermo Fisher Scientific Inc. (Waltham, MA, USA). Eu-W1024-anti-6xHis antibody (cat. #AD0400) and 384-well white ProxiPlates^TM^ (cat. # 6008289) were purchased from Perkin Elmer, Inc. (Waltham, MA, USA). Echo-qualified 384 well COC low dead volume source microplates (cat. #001-16128) and Echo-qualified 384 well polypropylene microplates (cat. #001-14615) were purchased from Beckman Coulter Inc.(Indianapolis, IN, USA). The assay was carried out in an enclosed workcell with subdued lighting. All reagents were prepared in the assay buffer (50 mM HEPES, 10 mM MgCl$_2$, 1 mM EGTA, 0.01% Brij-35, 1 mM DTT) and kept on ice. These included 2 x tracer 236 (0.2 $\mu$M), 2 x IRAK1 /antibody solution (20 nM IRAK1-His, 4 nM Eu-W1024-anti-6xHis antibody) and 2 x antibody solution (4 nM Eu-W1024-anti-6xHis antibody). Five microliters of 2 x tracer 236 was dispensed into a 384-well white ProxiPlate^TM^, followed by either 5 $\mu$l of 2 x IRAK1/antibody solution or 5 $\mu$l of 2 x antibody solution on a Tempest® dispenser (Formulatrix, Inc., Bedford, MA, USA). The plate was sealed on a Wasp plate sealer (KBiosciences Limited, Basildon, Essex, UK) and centrifuged at 1000 x g for 15 s on a HiG^TM^ automated centrifuge (BioNex Solutions Inc., San Jose, CA, USA) and incubated at room temperature for 30 min. The plate was then peeled and read on a PHERAstar® FSX (BMG LABTECH Inc., Cary, NC, USA) with a LanthaScreen^TM^ module at 340/615, 665 nm. The TR-FRET ratio (acceptor emission/donor emission x 10,000) was used as the readout.

***Biovalidation***

Biovalidation was carried out with identical assay settings as for the anticipated production runs. Assay conditions and the instrument settings were tested for their performance within the acceptance criteria. The acceptance criteria can be quantified by setting a minimum Z$'$ (see Eq. 1) to 0.5, where *p* and *n* refer to positive and negative control wells in the plates. Compounds from 2 library plates were dispensed at 10 nL/well in single point in columns 3 to 22 on assay plates (final concentration in assay at 10 $\mu$M) and 10 nL/well of DMSO was dispensed in columns 1, 2, 23 and 24 for controls. Ten nanoliter per well of DMSO was dispensed into all wells on positive and negative control plates. Control plates only have DMSO dispensed to all the wells. The measured difference in response is between the tracer (substrate) alone or the kinase with the tracer being dispensed in the wells to simulate fully inhibited enzyme or fully active enzyme.

Compounds and DMSO were dispensed on an Echo 655 liquid handler in an Access workstation. For the kinase binding assay, the 2 x tracer solution was dispensed into all wells on all plates. For the assay plates, the 2 x IRAK1/antibody solution was dispensed into columns 1 and 3 to 23. The negative control plates have the same layout as the assay plates, with DMSO in place of the compounds. For the positive control plates, the 2 x antibody solution was used in place of the 2 x IRAK1/antibody solution in columns 3 to 22. Six plates were dispensed in total, including 2 assay plates, 2 negative control plates and 2 positive control plates. The compound dispense run and the binding assay run were both set up and launched in the Cloud Lab. The automated runs were carried out in the workcells, and with the autoprotocols designated for production. Z$'$, signal-to-background ratio and compound hit rate were analyzed as performance parameters.

1 $$\begin{aligned} \text {Z}' = 1 - \frac{3(\sigma _p + \sigma _n)}{|\mu _p - \mu _n|} \end{aligned}$$

***Pilot screen***

Biovalidation was followed by a pilot screen with a plate number close to that in a production run for evaluation of the robustness of the assay, the automation scheduling and the data transfer. Compounds from 20 library plates were dispensed onto 20 assay plates. Two positive and two negative control plates were used in the same manner as in biovalidation. The screen was carried out with the same lot of reagents, procedure, instrument settings and autoprotocols as in biovalidation. Z$'$, signal-to-background ratio and compound hit rate were analyzed as performance parameters. No issues were observed in the pilot screen and the primary screen could be commenced.

### High-throughput screen (HTS)

***Primary screen***

The primary screen runs were performed with the same reagents and procedures as the pilot screen. Up to 40 plates were assayed per run. In total, 153 plates and 46,743 compounds were screened at 10 $\mu$M in single point. Plate quality control was performed using manual inspection and $Z'$ analysis (Eq. 1). Plates not passing with $Z'\ge 0.5$ were re-run. Note that in the first run, 3 of the 153 plates did not satisfy $Z'\ge 0.5$ ( 2%). These 3 plates were all repeated and subsequently satisfied the aforementioned criteria, such that all 153 plates ultimately passed the $Z'\ge 0.5$ threshold.

We normalized the fluorescence data on a per-plate basis using the collected fluorescence measurements. Normalization used both negative (DMSO) and positive (Staurosporine) controls to scale the fluorescence in the ratio channel (see Eq. 2). Across each plate, mean values of the 32 negative control ($\mu _{DMSO}$), and 32 positive control ($\mu _{SS}$ - Staurosporine) wells were used to normalize the raw ratio channel $k_{raw}$. Normalized values represent the relative inhibition of IRAK1, where 0% corresponds to the negative control - no inhibition, and 100% corresponds to the positive control - inhibition to the level of staurosporine.

2 $$\begin{aligned} k_{norm} = \frac{k_{raw} - \mu _{DMSO}}{\mu _{SS} - \mu _{DMSO}} \end{aligned}$$

**Fig. 2:**
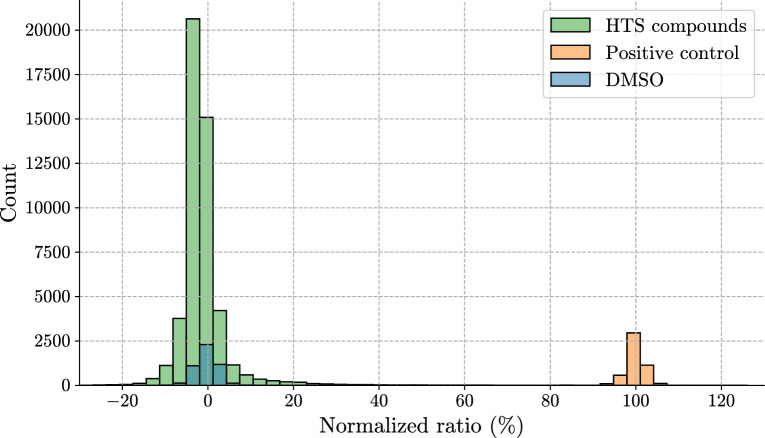
Normalized fluorescence values in the ratio channel from the IRAK1 HTS. Distributions from fluorescence values obtained from compounds in the diversity library, as well as the corresponding positive and negative control, are represented in different colors. Here, 0% corresponds to the mean normalized fluorescence ratio in negative control wells, and 100% to normalized fluorescence ratio in positive control wells across the whole library. Positive control represents IRAK1 inhibition with staurosporine

The distribution of normalized fluorescence ratio values is presented in Fig. 2. Only normalized fluorescence ratio channel values were used in further analysis. The arbitrary threshold of 50% normalized fluorescence ratio was chosen for hit selection based on the approximate number of hits that could be considered for secondary assay. Using this threshold, 353 hit compounds were identified.

***Single-dose hit confirmation***

We performed a single-dose hit confirmation in triplicate to evaluate data consistency in the primary assay. Top-10 plates with the highest hit count were re-run in additional duplicate experiments. In these plates there were 94 hits in total, 88 of which were confirmed and no additional hits discovered, constituting a precision of 93.4% and recall of 100%. The experiments were of high consistency and quality, with Z$'$ values above 0.6 for all plates, and high correlation in normalized fluorescence ratio values between the pairs of replicates ($\ge 0.8$).

***Compound clustering***

The 353 hits identified via HTS were subsequently clustered by their structural similarity using the Louvain algorithm [34]. The algorithm identifies clusters (“communities”) within a graph of related compounds that is constructed using compound Tanimoto similarity (TS) based on Morgan fingerprints. The Louvain algorithm was chosen for its compatibility with Tanimoto similarity and robustness to the number of clusters in the dataset. In total, 200 unique clusters were identified, 160 of which were singletons. Five compounds with the greatest ligand efficiency (LE) values were selected from each cluster to form a diversified set of 283 hits. A proxy for ligand efficiency was used, computed by dividing the normalized ratio value by the molecular weight of the compound.

***Hit dose-response assay***

A dose-response assay was conducted for each of the compound in the diversified set of 283 hits. Each compound was assayed in an 8-point curve with approximately 4-fold dilutions (subject to Echo dispense volume limits), starting at 30 $\mu$M, and the assays were run in triplicates. The exact concentrations are 30, 7.5, 1.875, 0.469, 0.117, 0.029, 0.007, 0.002 $\mu M$. In each plate, three replicates of a staurosporine titration curve starting at 3 $\mu$M were assayed in parallel as a reference.

The IC$_{50}$ of each dose-response curve was derived by fitting a four-parameter logistic (4PL) model, shown in Eq. 3, where the respective variables are defined as follows:

- $A$: Minimum asymptote. Response value when $x$ approaches infinity.
- $D$: Maximum asymptote. Response value when $x$ is very small or close to zero.
- $B$: Slope factor (Hill’s slope). Steepness of the curve.
- $C$: Inflection point. The concentration of the analyte that gives half-maximal response.

3 $$\begin{aligned} f(x) = A + \frac{D - A}{1 + \left( \frac{x}{C}\right) ^B} \end{aligned}$$

The 4PL model was fitted for each compound with data points for all three replicates all at once. As an additional quality control, 4PL regression models for all sub-micromolar compounds were manually inspected. Computed IC$_{50}$ were capped within the range of measured concentrations. In seven cases, where curve fits were erroneous and produced IC$_{50}$ values above assay sensitivity range, IC$_{50}$ values were reduced to the highest concentration used in the assay (30 $\mu$M).

**Fig. 3:**
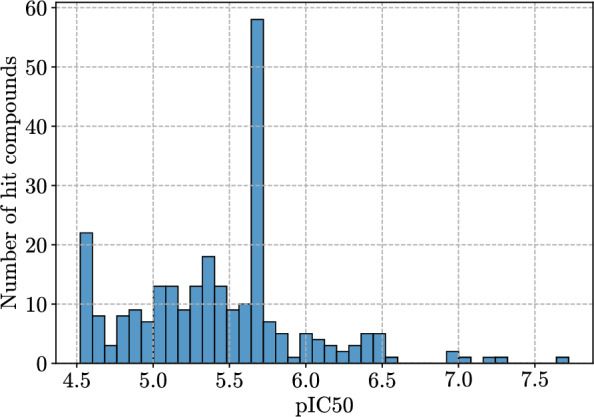
Distribution of pIC$_{50}$ values across the diversified set of 283 hits

The resulting pIC$_{50}$ distribution is presented in Fig. 3. Note that the distribution contains two peaks, one at $\sim 4.52$ pIC$_{50}$ and another at $\sim 5.7$ pIC$_{50}$. The first peak is due to 30 $\mu$M being the highest concentration and the fitting process described before. The second peak is an experimental artifact: due to the lack of assay resolution in between 1.9 $\mu$M and 7.5 $\mu$M, multiple model fits produced the same IC$_{50}$ value (1.9 $\mu$M).

## Results

### Target evaluation using SpectraView

Multiple protein targets were considered for the joint Ro5-Strateos project. The targets were proposed by Strateos based on the availability of scalable assays and interest from collaborators. We employed SpectraView to perform a thorough assessment of each target and identify one that is therapeutically relevant, commercially viable and could also be used for the prospective validation of HydraScreen. SpectraView relies on Ro5’s integrated Knowledge Graph to serve information from multiple data sources (see methods section 2.1) following these consideratons:

- Availability and quality of the crystal structure(s)
- Existing biochemical assay data
- Existing drugs and potent compounds
- Publication count and trends
- Novelty/Traction balance
- Target-disease associations
- Translation from academia to industry
- Competitive landscape

**Table 1:** Targets considered for the Ro5-Strateos project with a subsample of the corresponding data used in target evaluation. Data was extracted from RSCB PDB [35], PubChem [36] and DrugBank [37] at the start of the project (January 2022)

Target	Crystal structures	Assay Data Points (1000s)	Max. affinity (nM)	FDA approved drugs
JAK1	44	6.5	$<0.01$	5
JAK2	115	10.0	$<0.01$	5
JAK3	38	6.0	$<0.001$	5
TYK2	38	3.5	$<0.7$	1
IRAK1	1	1.3	$<5.6$	0 / 1 inv.$^a$
FGFR1	59	7.0	0.2	5
FGFR2	37	2.1	0.1	7
FGFR3	4	4.5	0.1	9
FGFR4	28	2.0	0.1	6
RIPK2	24	0.2	1.3	0 / 1 inv.
VGFR2 (KDR)	45	18.0	0.02	2
TAK1 (MAP3K7)	19	0.3	1	-

$^{a}$ Early clinical studies of IRAK1 inhibitor R835 [38]

The desired availability and quality of the crystal structures were achieved through a combination of selecting high-resolution X-ray crystallographic structures (with resolutions below 2.5 Å) and prioritizing holo-conformations (structures of targets bound with ligands). One of the main criteria when selecting a target is its novelty/confidence trade-off [9]. We have assessed the novelty of a target by using information on PubMed-indexed publications, availability of crystal structures, biochemical assay data, and approved or investigational drugs (Table 1). Most of the considered targets are very well-studied, as marked by the volume of PubMed publications mentioning them (e.g.  800 articles published each year that mention KDR, see Appendix Fig. A1). We focused on the less established targets with lower volume of publications, fewer data points and only few known high activity compounds - IRAK1, FGFR3 and TAK1.

The availability of a crystal structure was a crucial consideration when selecting a target for the prospective validation of HydraScreen. The crystal structure is necessary to generate ligand poses in the protein binding site which are then used by HydraScreen to predict ligand affinity and pose confidence scores. Additionally, we have assessed the availability of assay data, which could be used as a reference to compare HydraScreen with QSAR-based machine learning models. All of the considered targets had at least 1 crystal structure (Table 1). The crystal structure of IRAK1, one of the least established targets, was only recently resolved [39] (6BFN, 2.23 Å). Moreover, 1.3*k* biochemical assay data points were available for IRAK1, that could be used in training a QSAR model. IRAK1 thus satisfied the minimal requirements, while also being the most underexplored target.

**Fig. 4:**
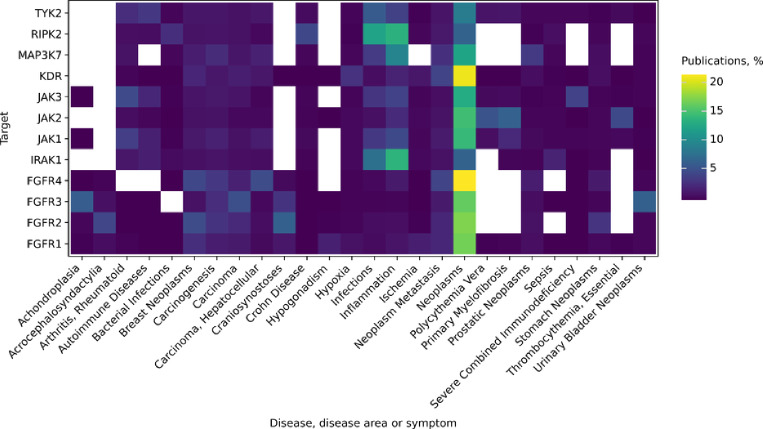
Diseases, disease areas and symptoms co-mentioned with each of the considered targets. Colors represent the fraction of PubMed-indexed publications per disease for each of the targets

Additional evidence was needed to substantiate IRAK1’s choice in terms of its therapeutic links. In contrast to many other kinases, IRAK1 is primarily associated with inflammation (Fig. 4, e.g. [40]), rather than cancer. It is only recently that IRAK1 has been linked to multiple cancers, including breast cancer [41], lymphoma [42] and acute myeloid leukemia [43]. The combination of fewer publications and emerging new therapeutic links provided additional support for IRAK1’s selection.

Finally, IRAK1 was assessed in terms of the potential competitors in the drug development field. We conducted an analysis of the competitive landscape by querying the publications and patents held by major pharmaceutical companies, as well as the most potent drugs and compounds reported in the public domain. We identified a limited number of PubMed-indexed publications with affiliations linked to major pharmaceutical companies: Johnson and Johnson - 4, Genentech - 2, Roche - 2, GlaxoSmithKline - 2, Pfizer - 2, Novartis - 1, Rigel - 2 (Suppl. Figure A3. Additionally, in comparison to other targets in consideration, IRAK1 had relatively fewer publications with industry versus academia affiliations (Suppl. Figure A5). The industry versus academic publication ratio could be interpreted as a proxy of the translation of basic research to drug development for a given target. IRAK1 was below the trend observed for other targets, thus potentially indicating its lower relative translation. Similarly, we assessed patents and patent applications (Suppl. Figure A4). The majority of patents or patent applications mentioning IRAK1 were owned by two academic instiutions - Dana Farber Cancer Institute and Yissum Research and Development Company of the Hebrew University, with each of these holding 14 patents. No major pharmaceutical companies (e.g. AstraZeneca, GlaxoSmithKline, Novartis, Sanofi) were found to hold patents linked to IRAK1.

Finally, we assessed the chemical matter linked to IRAK1 - the most potent compounds and drugs targeting it. Only a few high-affinity compounds have been reported for IRAK1 (42 with pIC$_{50}$>7 and 2 with pIC$_{50}$>8, e.g. JH-X-119-01 with 9 nM affinity, [42]). Currently there are no FDA approved drugs that would target IRAK1. Rigel Pharmaceuticals has recently started pre-clinical and clinical studies of IRAK1/4 inhibitor R835, demonstrating potential in murine models for multiple inflammatory diseases, including arthritis and lupus. However, this compound has not yet received an FDA approval [38]. An active metabolite R406 of an FDA approved drug Fostamatinib has been shown to have an off-target affinity for IRAK1 [44]. Fostamatinib was also developed by Rigel Pharmaceuticals for the treatment of chronic immune thrombocytopenia. The combination of largely academic research in IRAK1 with only recently emerging interest by pharmaceutical companies (Suppl. Figure A5), especially the supporting pre-clinical and clinical work [38, 44], provides corroborative evidence for its potential as a prospective drug target. The lack of any FDA-approved drugs targeting IRAK1 leaves an opportunity for the development of novel small molecule inhibitors. Altogether, the novelty/confidence trade-off balance, sufficient support in terms of biochemical and biological rationale as well as competitive considerations made IRAK1 an attractive target to be pursued in this study.

### Identification of IRAK1 hits using HydraScreen

#### HydraScreen virtual screen

Following the selection of IRAK1 using SpectraView, we performed *in silico* virtual screening and experimental hit identification via HTS. The goal of this stage of the project was to prospectively evaluate HydraScreen’s [25] performance using *in vitro* data collected by Strateos’ HTS and compare it against traditional, industry-standard methods including *Smina* [17] (molecular docking), *DeCAF* [29] & *Pharmit* [45] (pharmacophore modeling) and a RF model trained on publicly available IRAK1 assay data (QSAR modeling). These findings collectively provide a comprehensive and unbiased evaluation of HydraScreen as a virtual screening method.

Strateos 47k compound library was screened using HydraScreen, as described in methods section 2.5. Affinity predictions were used to rank the compounds and select the top 1% (470) to be considered as *in silico* hits. Strateos subsequently performed an *in vitro* primary assay HTS using the same library. HTS identified 353 hit compounds at the 50% normalized fluorescence ratio threshold. Note that 359 hits were originally found. However, 6 of these were subsequently removed following the results from triplicate experiments. A 50% threshold was chosen because it filtered out sufficient compounds to reach the desired number of candidates that could be validated in the secondary assay, with a surplus to account for potential compound detrition. These compounds were compared to the ones ranked in the top 1% by HydraScreen. In total, HydraScreen discovered 57 hits that were also identified in the HTS, constituting a 15.9% hit discovery rate via virtual screening (see Supplementary Table available with the pre-print).

**Fig. 5:**
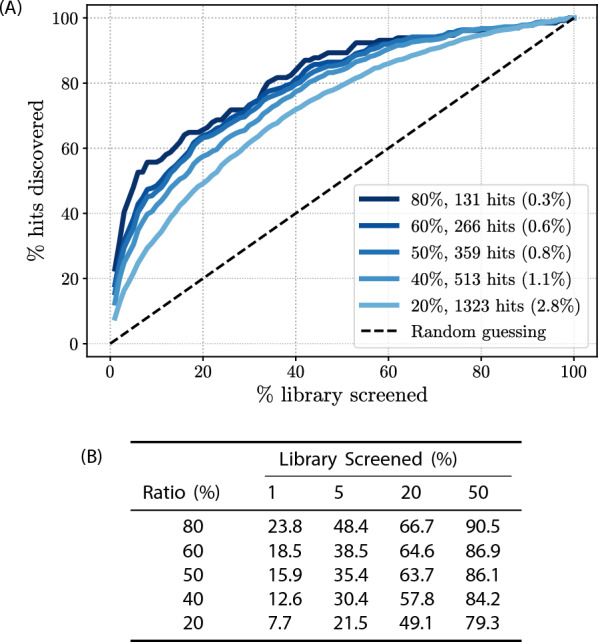
**A** HydraScreen hit discovery rate (% of hits discovered per library screened) for different IRAK1 inhibition thresholds in HTS (ratio %, marked by lines of in the shades of blue). For each IRAK1 inhibition threshold the number of hits identified in HTS is presented together with the overall HTS hit rate. Dashed black line represents random compound ranking. Supporting data is presented in table **(B)**

We next investigated the impact that different normalized fluorescence ratio thresholds used for hit selection in HTS can have for hit identification in the HydraScreen virtual screen (Fig. 5). As both virtual in silico and high-throughput in vitro screens rely on arbitrary thresholds for hit selection [12, 13], it is important to understand the model performance under a range of such thresholds. Here, we considered the comparison of virtual screening predictions against the HTS results for each individual compound in the ranking generated by HydraScreen. Virtual screening hit recovery rate for HydraScreen is estimated as the proportion of hits identified per number of compounds in the corresponding library rank. Standard HTS protocols randomly test compounds from the library (i.e. in the order in which they are stored); therefore, the hit recovery rate of traditional HTS is roughly proportional to the percentage of the library screened (diagonal dashed line in Fig. 5A). Any method that is able to prioritize active compounds over the inactive ones would provide a better hit recovery rate than random sampling (i.e. above the dashed diagonal line in Fig. 5A).

We find that ranking the compound library according to HydraScreen’s predictions greatly increases hit discovery rates. This result is also consistent for any proportion of compounds selected in the ranking, as well as for any relative inhibition fluorescence threshold. Using the 50% IRAK1 inhibition threshold, as was used in the *in vitro* experiment, HydraScreen identified 35.4% of the hits within the top 5% and 63.7% within the top 20% of the ranking (Fig. 5B). Notably, close to 90% of the hits can be identified within the top 50% of the ranked compounds (see Fig. 5B). HydraScreen exhibits better performance at higher IRAK1 assay normalized fluorescence ratio thresholds. For example, HydraScreen identified 23.8% (30 out of 126) of hits at the top 1% of the compound ranking when using 80% relative inhibition threshold of IRAK1 (Fig. 5B).

We next assess HydraScreen’s performance in terms of its ability to prioritize highly active compounds that are also structurally diverse. The number of distinct highly active scaffolds identified in HTS can often be a more relevant metric in drug discovery campaigns than the raw hit rate: greater variety of scaffolds provides medicinal chemists with more opportunities for lead series development, which is crucial at the later stages of drug discovery [46]. Moreover, high diversity of the identified hits increases the likelihood of discovering novel scaffolds which do not overlap with existing patents.

**Fig. 6:**
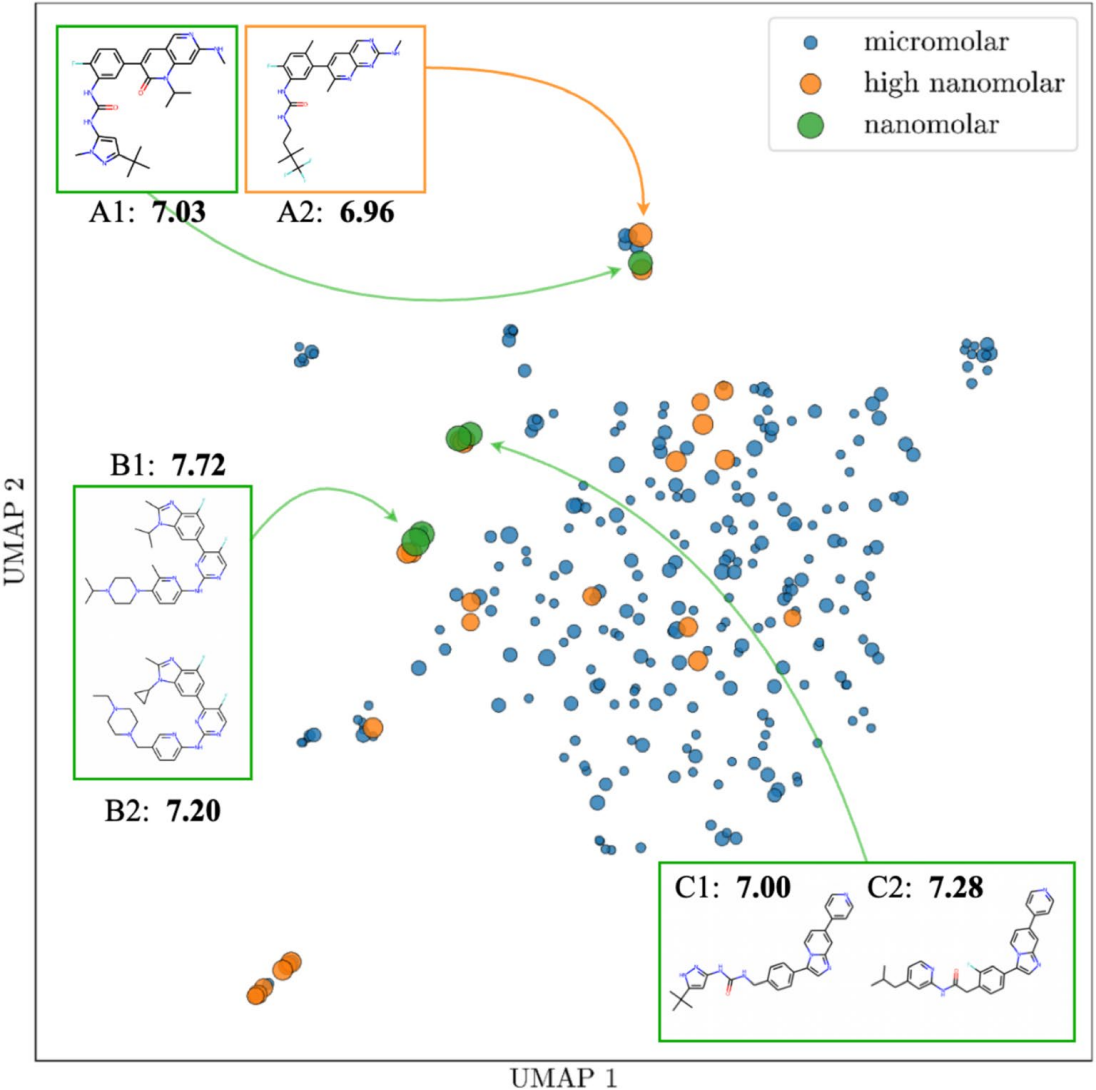
2D Uniform Manifold Approximation and Projection for Dimension Reduction (UMAP) [47] projection of the ECFP4 embeddings for 283 hit compounds from HTS screen. The space in the plot represents relative similarity of the compounds. Nanomolar compounds from the three nanomolar scaffolds are highlighted with their $pIC_{50}$ values indicated underneath. Marker size is proportional to compound activity. More details about the nanomolar compounds are given in Table B1

In order to conduct the secondary assay and identify IC$_{50}$ values we performed further assessment of hits. We selected a diverse, representative and unbiased set of compounds to be screened in the secondary assay by clustering the 353 hits from HTS according to their structural similarity using the Louvain algorithm [34]. In total, we extracted 200 unique clusters, 160 of which belong to single compound members. We identified core scaffolds within each cluster via maximum common substructure (MCS) analysis and select five compounds with the greatest ligand efficiency (LE) from each cluster to form a diversified set of 283 hits, each originating from 200 distinct scaffolds. For these 283 diversified hit compounds, we collected dose-response data (see methods 2.8). Based on their pIC$_{50}$ ($-log_{10}(IC_{50})$) activity values, hits and their corresponding scaffolds are grouped into micromolar, high nanomolar and nanomolar groups (Table 2). We identified 5 nanomolar and 25 high nanomolar hits, while the rest possessed micromolar activity (Fig. 6). Scaffolds were labeled based on the most active compound in each cluster. Out of the 200 defined scaffolds, 15 were labeled as high nanomolar and 3 as nanomolar. We refer to the union of high nanomolar and nanomolar compounds as *sub-micromolar*.

**Table 2:** Dose-response assay results for 283 diversified hits. Compounds and scaffolds were labeled as micromolar, high nanomolar and nanomolar based on the their pIC50 values. For scaffolds, the highest activity found in the corresponding cluster of compounds was used as a label

Range	pIC50	Compounds	Scaffolds
Micromolar	< 6	253	182
High nanomolar	6 $\le$ x < 7	25	15
Nanomolar	$\ge$ 7	5	3

**Fig. 7:**
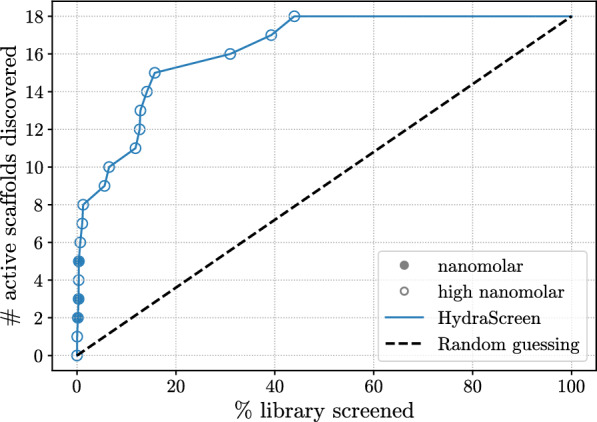
HydraScreen distinct scaffold discovery rate (number of distinct scaffolds discovered per library screened). Dashed black line represents random compound ranking. Filled and empty circles represent nanomolar and high nanomolar scaffolds, respectively

We used the dose-response data to evaluate HydraScreen’s performance in terms of discovery of highly active scaffolds (Fig. 7). We considered a scaffold “discovered” by a model if at least one compound from the corresponding cluster is ranked by the model in the corresponding top rank of the library. Notably, HydraScreen successfully ranked compounds belonging to all 3 nanomolar scaffolds within the top 1% of the library. Within the top 2%, HydraScreen ranked 8/18 of the submicromolar scaffolds. The remaining 10 scaffolds were ranked in the top 50% of the ranked compounds.

#### HydraScreen comparison against other virtual screening techniques

**Fig. 8:**
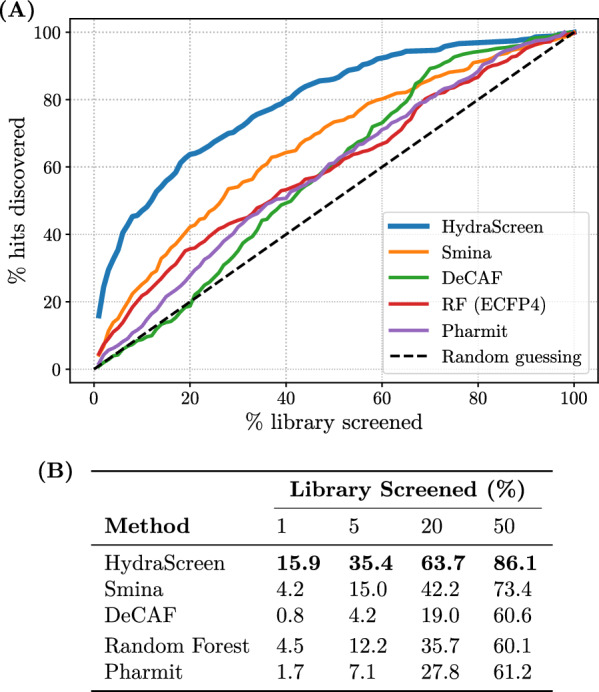
**A** Hit discovery rates provided by different methods in IRAK1 virtual screen. Dashed black line corresponds to random compound ranking. Supporting data is presented in table **(B)**

Virtual screening can be performed using a range of different techniques [48]. It is therefore relevant to evaluate HydraScreen’s performance in comparison to different traditional methods. In parallel to the HydraScreen virtual screen, we also prospectively generated predictions via SBDD through docking with Smina [17], a fork of AutoDock Vina with additional functionalities, shape similarity via 2D (DeCAF) and 3D (Pharmit) pharmacophore matching, and a QSAR-based RF model trained on molecular fingerprints (see Methods 2.6). Note that an exhaustive benchmark across additional industry-standard SBDD methods such as Gold [49] or Glide [50, 51] is out of the scope of this prospective study. Particularly, based on previous studies, traditional physics-based SBDD approaches frequently report similar overall performances in identifying hits in HTS [52, 53] and assessing protein-ligand affinities [25, 54].

We selected a hit pool based on the 50% IRAK1 normalized fluorescence ratio threshold used in primary assay, with 353 hits identified in total, and measure the hit discovery rates obtained across each method (Fig. 8). Notably, HydraScreen significantly outperforms other techniques, consistently achieving higher hit identification rates across different selections of top ranked compounds. At the top 1% ranking, the model provides 3.5x better performance than traditional docking, 3.2x higher EFs than ML-based QSAR models, and $\sim$20-fold higher rates compared to shape-based similarity methods (Fig. 8B).

**Fig. 9:**
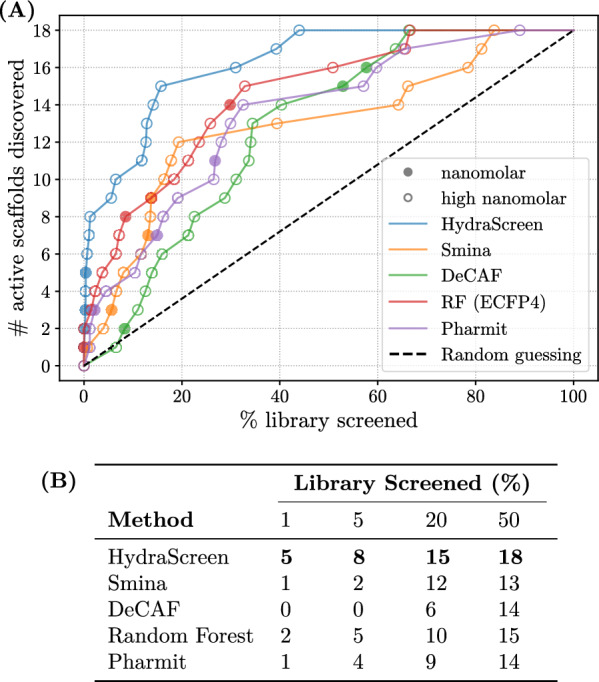
**A** Scaffold discovery rates provided by different methods in IRAK1 virtual screen. Nanomolar and high nanomolar scaffolds are marked by filled and empty circles respectively. Dashed black line corresponds to random compound ranking. Supporting data is presented in table **(B)**

We also assessed the ability to identify diverse chemical scaffolds across the aforementioned virtual screening methods. As previously outlined in HydraScreen’s scaffold recovery analysis, we considered a scaffold to be “discovered” if at least one compound from that scaffold is selected within the corresponding screening range. We present our findings in Fig. 9. Similar to the increased hit rates observed in Fig. 8, HydraScreen exhibits superior scaffold discovery rates. Within the top 1% of the library, HydraScreen ranked all three nanomolar scaffolds and, in total, 6 out of 18 submicromolar scaffolds (Fig. 9B). In comparison, Smina ranked the last nanomolar scaffold at 18%, Pharmit at 27% and RF at 30%. Moreover, Random forest QSAR model ranked one of the nanomolar scaffolds in the top 10 compounds (0.02%). However, this scaffold is a direct analogue to a compound present in an IRAK1 assay data which the RF model has trained on, reflecting on its ability to internalise a non-linear similarity search, rather than generalising protein-ligand affinity prediction. More details on this particular scaffold and the analogue will be discussed in Sect. Hit novelty and properties.

### IRAK1 hits

In IRAK1 HTS we discovered 353 hit compounds out of which a diversified set of 283 compounds corresponding to 200 distinct scaffolds was selected for secondary assay. In the last stage of the project, we evaluate these compounds and scaffolds in terms of their novelty, physico-chemical properties and IRAK1 binding modes.

#### Hit novelty and properties

In order to assess the uniqueness of the 283 diversified hits, we compared them against IRAK1 actives available in PubChem. Out of the 689 compounds reported to be active against IRAK1, 141 have sub-micromolar activity. For each of the 283 hits, we found the nearest neighbor in the set of IRAK1 actives and scaffolds based on their Tanimoto Similarity (TS). The number of neighbors above a certain similarity threshold is reported in Table 3. We observe that the vast majority of hits are distinct from publicly known actives. Only 21 compounds, corresponding to 13 distinct scaffolds, exhibit >0.4 TS. In the nanomolar range, only 1 of the 3 distinct scaffolds have a similar active compound in the public domain; the closest structure is the Pan-RAF inhibitor LY3009120 [55] with a TS of 0.82. LY3009120 displays some IRAK1 inhibition (390 nM IC$_{50}$) in a whole cell-based kinase screen, however it is not the primary target of the compound.

**Table 3:** Numbers of hits and scaffolds that have at least one neighbor in the IRAK1 public dataset that is more similar than the specified Tanimoto similarity (TS) threshold

TS threshold	Hits	Scaffolds	Nanomolar scaffolds
None	283	200	3
> 0.4	21	13	1
> 0.6	5	3	1
> 0.8	1	1	1
> 0.9	0	0	0

We next investigated the structural diversity and physico-chemical properties of the most potent hits. The 30 sub-micromolar hit compounds represent 18 distinct scaffolds, with the six most active compounds spanning three of these as indicated in Fig. 6 by A, B and C. The six most active compounds are synthetically tractable, with synthetic accessibility scores in a similar range to that of catalogue compounds (2–3) [56]. They border on the upper end of the Lipinski rule of 5 [57] with regards to molecular weight (466 to 521 g/mol) and Crippen LogP values of 4.7 to 6 [58]. Their high molecular weight and hydrophobicity will have to be further assessed during a medicinal chemistry program.

#### HydraScreen hit compound binding modes

**Fig. 10:**
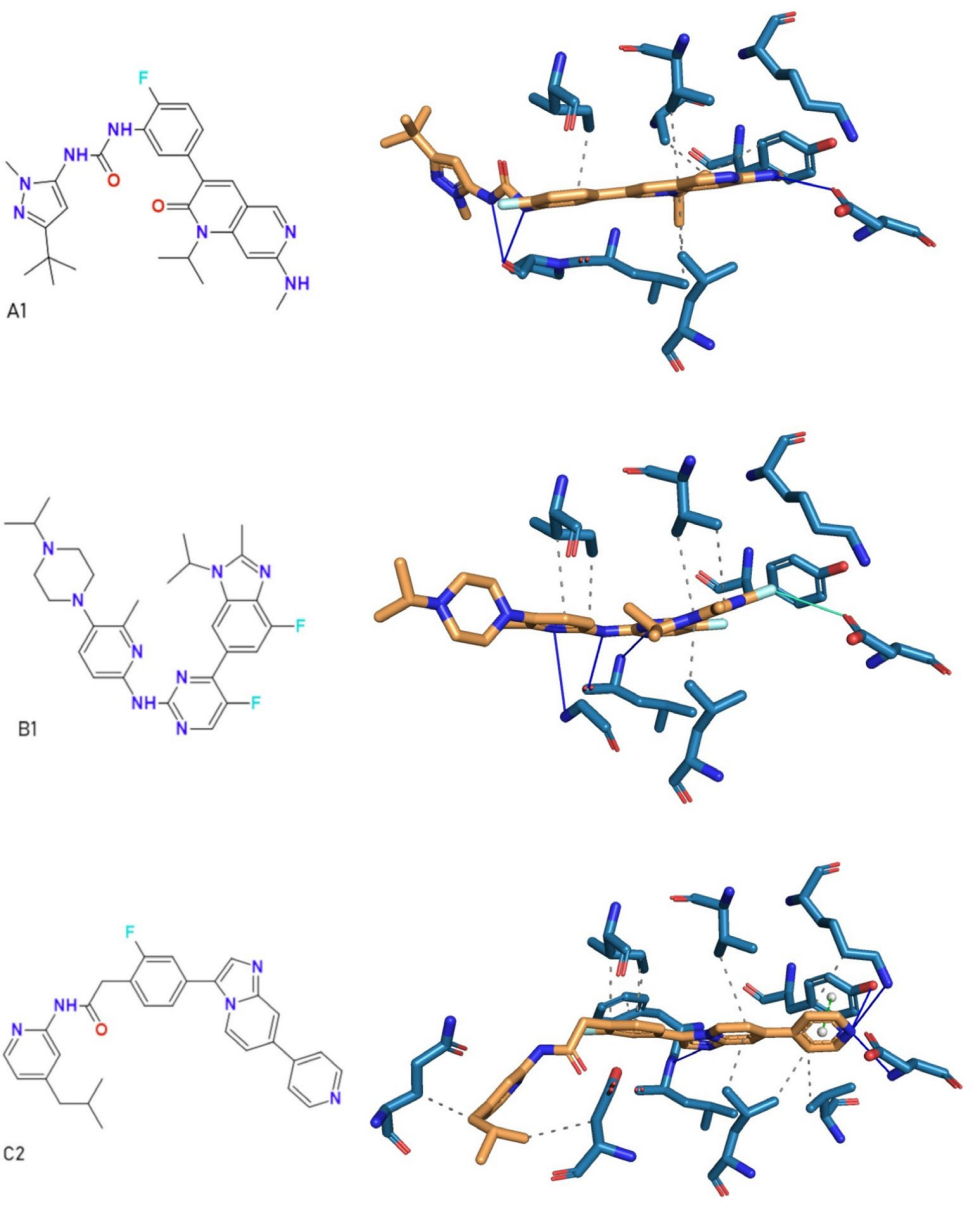
IRAK1-ligand poses with the highest HydraScreen confidence for selected nanomolar hits A1, B1, and C2. PLIP protein-ligand interactions are shown with grey dashes (hydrophobic interactions), blue lines (H-bonds), cyan line (halogen bond), and green dashes connecting white spheres (pi-pi stacking)

HydraScreen provides insight into the likely binding modes of the compounds by predicting ligand pose confidence scores. We investigated the binding modes for the highest confidence poses from each of the nanomolar scaffolds (compounds A1, B1, and C2 in Fig. 6). The IRAK1-ligand interactions for these poses were assessed using PLIP profiler [59]. Across the highest confidence poses, the sequential aromatic heterocycles of the compounds were situated towards the back of the ATP binding pocket, with hydrophobic interactions with valine (V226), leucine (L347), and isoleucine (I218) residues (Fig. 10). The central heterocycles of compounds B1 and C2 form hydrogen bonds (H-bonds) with the hinge region, whereas the urea in A1 forms H-bonds to the backbone. Both A1 and B1 interacts with the carbonyl of aspartic acid D358 in the back of the pocket, respectively through an H-bond and halogen bond. On the other hand, the highest confidence pose of compound C2 highlights a pi-stacking interaction with the gatekeeper residue tyrosine Y288, as well as H-bonds to both Y288 and the catalytic lysine K239. Across the compounds, aliphatic sp3-rich motifs are situated toward the solvent exposed region of the pocket.

Insights gained from HydraScreen regarding the compound poses and the different interactions of scaffold motifs aids further compound design by highlighting areas and interactions to exploit not only around a specific scaffold, but also from one scaffold to another. The hit compound activity, novelty, and ample positions to tailor, render them attractive scaffolds for further structure-activity relationship (SAR) exploration and subsequent hit-to-lead development.

## Discussion

***Accelerated hit discovery in IRAK1***

In this study, we propose an augmented drug discovery workflow that relies on Ro5’s AI and data science platform while utilizing Stateos’ robotic labs capabilities. We show how target evaluation driven by SpectraView guided the selection of IRAK1 serine-threonine kinase target. In comparison to other considered targets IRAK1 exhibits favorable novelty/confidence balance with relatively low number of publications from pharmaceutical companies and assay data points. Currently there are no FDA approved drugs targeting IRAK1 and only a few highly active compounds [42]. At the same time, emerging support for IRAK1’s therapeutic links to cancers and inflammation with recent pre-clinical and clinical work make it an attractive target to pursue.

We provide compelling evidence for HydraScreen’s virtual screening performance. Notably, HydraScreen exhibits high hit discovery rates in IRAK1 virtual screening, with upwards of 15.9% hits and all of the 3 nanomolar scaffolds identified within the top 1% of the compound library. HydraScreen also successfully ranked all of the distinct nanomolar and high nanomolar scaffolds in the top 50% of the compound library. Moreover, HydraScreen’s performance increases with stricter thresholds for experimental hit selection, where up to 23.8% hits were found within top 1% of the ranked compounds when using a relative inhibition threshold greater than 80%. Thus, HydraScreen successfully prioritizes highly active compounds and does not exhibit structural biases.

The prospective evaluation of HydraScreen has shown it to be superior to traditional, industry-standard methods like Smina, DeCAF and a QSAR RF model, in both hit and scaffold discovery. These results support previous *in silico* benchmarking results where HydraScreen exhibited state-of-the-art performance in line and above of the most recent AI models available for protein-ligand binding affinity prediction [25]. Importantly, HydraScreen training set does not include IRAK1 data, so these results also reflect on the model’s ability to generalize to an unseen target.

This study successfully identified novel and potent IRAK1 inhibitors. One of the identified nanomolar scaffolds exhibits high similarity to a known Pan-RAF inhibitor LY3009120 [55], while the other two are novel when compared to known IRAK1 actives. The five most potent nanomolar hits represent three distinct scaffolds, which are synthetically accessible. The high molecular weight and lipophilicity of the most potent hits will have to be further explored during a medicinal chemistry program. HydraScreen uniquely provides ligand pose confidence scores [25], a valuable feature for assessing the binding modes and potential modifications of the most potent hits during hit-to-lead and lead optimization stages of a drug discovery program. The highest confidence poses of the nanomolar hits indicated multiple IRAK1-ligand interactions to draw on for structure-activity relationship (SAR) exploration, both around a single scaffold and between scaffolds.

The most important contribution of our work is the prospective validation of HydraScreen for virtual screening. We provide a robust assessment of HydraScreen by experimentally screening the entire 47k library and report a hit discovery rate of upwards of 15.9% for the top 1% (470) of tested compounds. In contrast, prospective validation studies usually test only a small fraction of the library compounds, well below 1%, a median of 44 compounds (401 studies) [13]. Such studies report median hit rates $\sim 11.8\%$ across all target classes (385 studies) and $\sim 9.6\%$ for kinases (67 studies) [13]. However, these hit rates are prone to bias due to a small test size. Only 21 studies have tested more than 470 compounds and they report a substantially lower median hit rate of $\sim 2.16\%$ [13]. Moreover, a similar virtual screening study in IRAK1 reported a 2.83% hit rate [60]. HydraScreen’s hit rate is in the top 10% rank of the prospective validation studies that test at least 470 compounds and well above the median reported for kinases regardless of the test size [60]. Furthermore, HydraScreen can achieve even higher hit rates of up to 23.8%, in top 10% of similar or greater test size and greater than the 3rd quartile (23.5%) reported in [13] regardless of the test size. HydraScreen’s evaluation at stricter IRAK1 inhibition thresholds is potentially more representative of its true performance due to a higher confidence in the hits selected from the assay (i.e. lower false-positive rate).

***Future work***

There results presented in this study provide several directions for future work. First of all, it would be interesting to explore the effect of HydraScreen model fine-tuning on its performance. It is very probable that we could achieve even better results by fine-tunign the HydraScreen with publicly available data for IRAK1 or other closely related kinases (e.g. IRAK2, IRAK3, IRAK4). This concept could also be extended to create an active learning system that integrates experimental result collection and model inference. Model could be fine-tuned with the data collected during the *in-vitro* screen. Generating model predictions, collecting in-vitro screening results for selected compounds and fine-tuning the model for the next round of prediction could potentially enable screening of vast datasets. Finally, we have identifed promising IRAK1 hit series with favourable characteristics that could be pursued in a drug discovery program.

***Conclusion***

This study provides compelling evidence for the effectiveness Ro5’s innovative tools, SpectraView and HydraScreen in early stage drug discovery. Using SpectraView target evaluation, we prioritize IRAK1 serine-threonine kinase with emergent therapeutic links in inflammation and cancers. By leveraging Ro5’s HydraScreen and Strateos’ automated labs, we show how AI-driven virtual screening with HydraScreen could offer high hit discovery rates and reduce experimental costs. In the top 1% of the ranked compounds, HydraScreen identified all three nanomolar classes, and almost a quarter of the total actives in the library at >80% relative inhibition of IRAK1. The unbiased, prospective evaluation of HydraScreen and comparison against industry-standard methods supports the reliability and robustness of our findings. Ro5’s SpectraView and HydraScreen provide innovative methods that can expedite the early stages of drug discovery.

## Supplementary Information

Supplementary file 1.

## Data Availability

SpectraView is available online free of charge at https://spectraview.ro5.ai/. HydraScreen is available online free of charge at https://hydrascreen.ro5.ai/. HydraScreen is also available with open-source license for non-commercial use as a Python package installable via *pip* from PyPi repository (https://pypi.org/project/hydrascreen/). The corresponding GitHub repository for this package is available at https://github.com/Ro5-ai/hydrascreen. The package can be used to replicate the screening following the methods description provided in the manuscript. Poses for all of the ligands from the 47k library are available at https://ro5-public.s3.amazonaws.com/47k_poses.zip. The full compound library, virtual screening and experimental data is available in the supplementary materials table.

## Abbreviations

AI: Artificial intelligence
CNN: Convolutional neural network
DL: Deep learning
HTS: High-throughput screening
LE: Ligand efficiency
MCS: Maximum common substructure
ML: Machine learning
MLSF: Machine learning scoring functions
PAINS: Pan assay interference compounds
QSAR: Quantitative structure-activity relationship
RF: Random forest
SBDD: Structure-based drug discovery
TS: Tanimoto similarity
UMAP: Uniform manifold approximation and projection [47]

## Footnotes

1 https://spectraview.ro5.ai/.

2 https://hydrascreen.ro5.ai/, https://github.com/Ro5-ai/hydrascreen.
