# Supplementary material for: Accelerated hit identification with target evaluation, deep learning and automated labs: prospective validation in IRAK1
Source: J Cheminform. 2024 Nov 14;16:127. doi: 10.1186/s13321-024-00914-0 (PMC11566907; doi:10.1186/s13321-024-00914-0)
Supplement: Supplementary file 1 [file 13321_2024_914_MOESM1_ESM.pdf]

Appendix A SpectraView

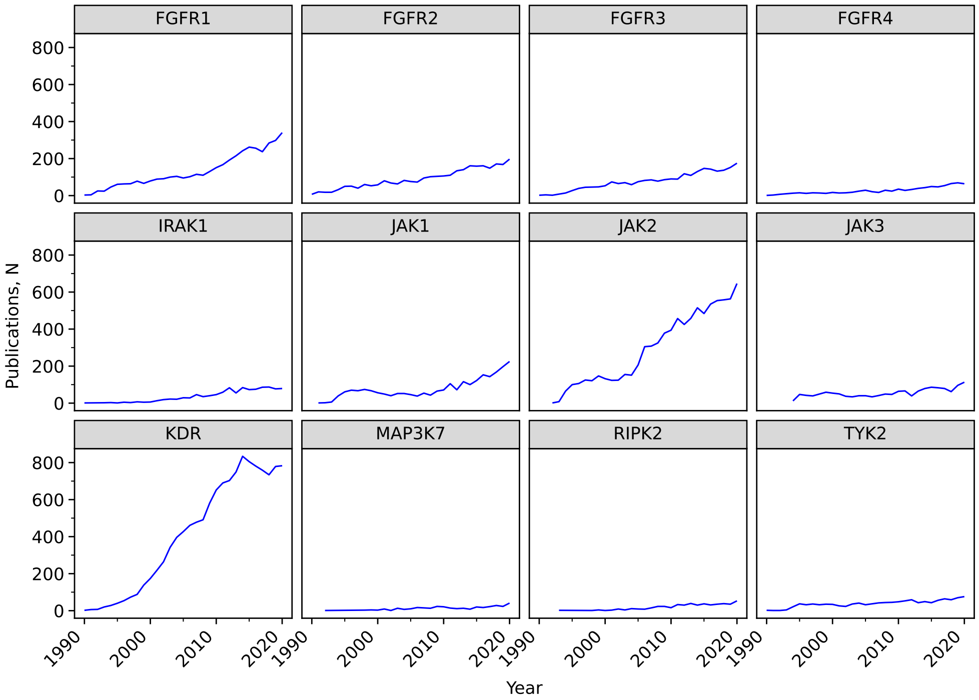

Fig. A1: PubMed-indexed publication trends over the last 3 decades for each of the considered targets.

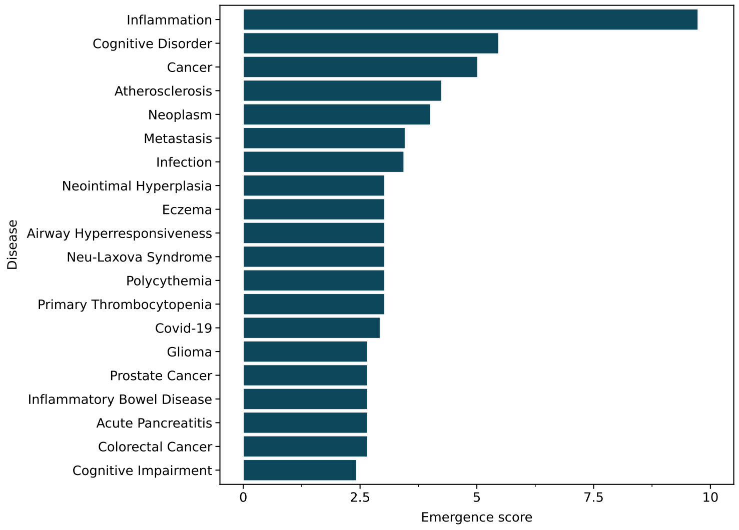

Fig. A2: Emergence scores (ES) for different diseases linked to IRAK1. The emergence score is calculated as a maximum increase in the number of publications per disease scaled by the total publication volume and recency.

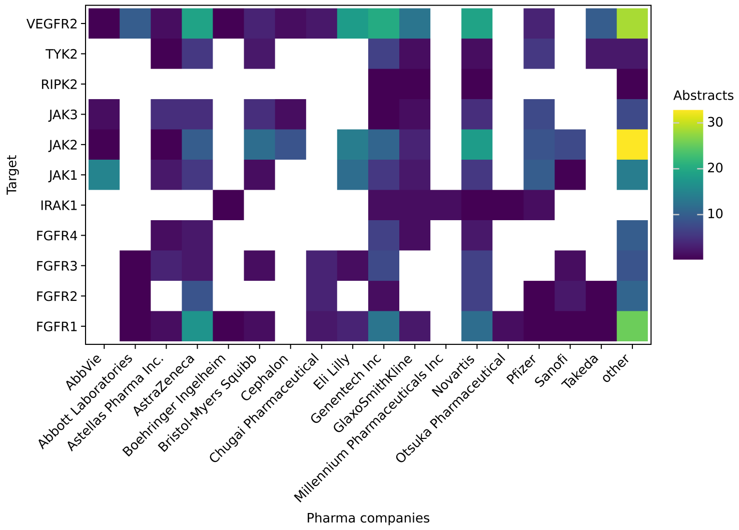

Fig. A3: Total number of PubMed-indexed publications with pharma company affiliations that at least one of the considered targets.

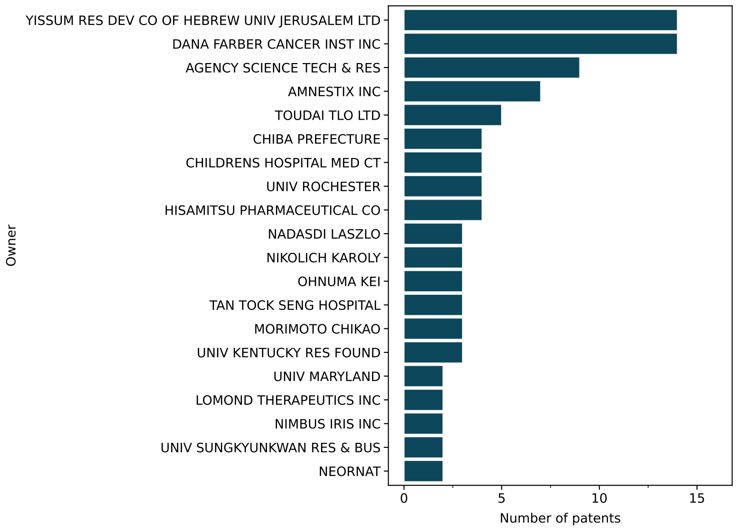

Fig. A4: Organizations that own the most patents or patent applications that mention IRAK1.

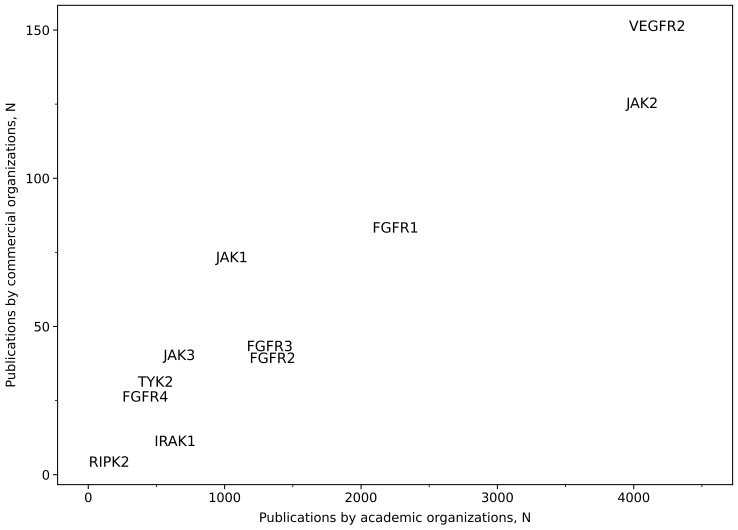

Fig. A5: The correlation between the number of PubMed-indexed publications affiliated with academic or pharma organizations for each of the considered targets.

Appendix B HydraScreen

Table B1: Detailed data on the nanomolar compounds visualized in the UMAP [48] plot in Figure 6. The data for the full library, including the HTS results and rankings of benchmark models, can be found in the supplementary material table.

Label	SMILES	DRC result, IC50 (micromolar)	DRC result, pIC50	DRC result, activity class	HydraScreen ranking
A1	CNc1cc2c(cn1)cc(-c1ccc(F)c(NC(=O)Nc3cc(C(C)(C)C)nn3C)c1)c(=O)n2C(C)C	0.09	7.04	nanomolar	305
A2	CNc1ncc2cc(-c3cc(NC(=O)NCCC(C)(C)C(F)(F)F)c(F)cc3C)c(C)nc2n1	0.11	6.96	high nanomolar	495
B1	Cc1nc(Nc2ncc(F)c(-c3cc(F)c4nc(C)n(C(C)C)c4c3)n2)ccc1N1CCN(C(C)C)CC1	0.02	7.72	nanomolar	1247
B2	CCN1CCN(Cc2ccc(Nc3ncc(F)c(-c4cc(F)c5nc(C)n(C6CC6)c5c4)n3)nc2)CC1	0.06	7.20	nanomolar	152
C1	CC(C)(C)c1cc(NC(=O)NCc2ccc(-c3cnc4cc(-c5ccncc5)ccn34)cc2)n[nH]1	0.10	7.00	nanomolar	1531
C2	CC(C)Cc1ccnc(NC(=O)Cc2ccc(-c3cnc4cc(-c5ccncc5)ccn34)cc2F)c1	0.05	7.28	nanomolar	291
